# Role of Vitamin D in Cognitive Function in Chronic Kidney Disease

**DOI:** 10.3390/nu8050291

**Published:** 2016-05-13

**Authors:** Zhen Cheng, Jing Lin, Qi Qian

**Affiliations:** 1 National Clinical Research Center of Kidney Disease, Jinling Hospital, Nanjing University School of Medicine, Nanjing 210016, China; chengzhen33@hotmail.com; 2 Division of Nephrology and Hypertension Department of Medicine, Division of Nephrology and Hypertension, Mayo Clinic College of Medicine, 200 First Street SW, Rochester, MN 55905, USA; lin.jing@zs-hospital.sh.cn; 3 Department of Nephrology, Zhongshan Hospital, Fudan University, Shanghai 200032, China

**Keywords:** vitamin D, hypovitaminosis D, cognitive impairment, chronic kidney disease, dialysis

## Abstract

Both vitamin D deficiency and cognitive impairment are common in patients with chronic kidney disease (CKD). Vitamin D exerts neuroprotective and regulatory roles in the central nervous system. Hypovitaminosis D has been associated with muscle weakness and bone loss, cardiovascular diseases (hypertension, diabetes and hyperlipidemia), inflammation, oxidative stress, immune suppression and neurocognitive impairment. The combination of hypovitaminosis D and CKD can be even more debilitating, as cognitive impairment can develop and progress through vitamin D-associated and CKD-dependent/independent processes, leading to significant morbidity and mortality. Although an increasingly recognized comorbidity in CKD, cognitive impairment remains underdiagnosed and often undermanaged. Given the association of cognitive decline and hypovitaminosis D and their deleterious effects in CKD patients, determination of vitamin D status and when appropriate, supplementation, in conjunction with neuropsychological screening, should be considered integral to the clinical care of the CKD population.

## 1. Introduction

Suboptimal vitamin D status, hypovitaminosis D (vitamin D insufficiency and deficiency), is extremely common [1]. Chronic kidney disease (CKD) has also become extremely common [2]. Both conditions, particularly prevalent in elderly patients (>65 years of age), are known risks for and closely associated with cognitive decline [3,4]. Although it has been hypothesized that CKD patients with inadequate vitamin D status could potentially experience an accelerated cognitive decline, there are scant adequately designed studies on this topic. In this review, we discuss vitamin D function in both healthy and CKD populations, centered specifically on vitamin D in brain function and the role of hypovitaminosis D in cognitive impairment (CI). We gathered the current evidence on occurrence rate, risk and role of hypovitaminosis D in non-dialysis and dialysis-dependent CKD patients with CI. We also provided clinical suggestions based on existing evidence for managing CKD patients with hypovitaminosis D.

## 2. Vitamin D Biogenesis, Metabolism and Function in Healthy and in CKD Patients

Vitamin D is acquired through diet, supplementation and photosynthesis. Two forms of vitamin D exist in circulation: 25(OH)D and 1,25(OH)_2_D. 1,25(OH)_2_D is the active form, generated from 25(OH)D through 1α-hydroxylation. The hydroxylation occurs primarily in the kidney but also in multiple non-renal tissues including brain [5,6,7]. In addition to its classic role in regulating calcium and phosphorous and maintaining bone health, vitamin D possesses a pleiotropic effect across multiple extraskeletal systems. Emerging evidence shows that vitamin D is able to regulate cellular proliferation and differentiation, immunity [8], inflammation [9], neuron health (detailed in Section 3) and the endocrine system including the renin angiotensin system (RAS), insulin secretion and lipid metabolism through interaction with intracellular vitamin D receptor (VDR) expressed in multiple systems [1,6,7,10]. Studies have also shown that vitamin D may exert protective effects against the development of type 1 diabetes mellitus [11], rheumatoid arthritis [12], multiple sclerosis [13,14], infection [15,16] and certain forms of cancer [17,18].

Vitamin D status is determined clinically by measuring the circulating 25(OH)D level [19,20]. The optimal level in the general population and CKD patients has not yet been firmly established. Consensus, based on bone health and mortality data, suggests that circulating 25(OH)D levels of >30 ng/mL (converted to nmol/L by multiplying 2.5 to the value of ng/mL) and <80 ng/mL are optimal [21,22]. Suboptimal vitamin D status is further grouped to insufficiency and deficiency (Table 1).

Suboptimal vitamin D is extremely prevalent worldwide and has become endemic. A meta-analysis by Chowdhury *et al.* shows that the prevalence of suboptimal vitamin D (insufficiency and deficiency) is 69.5% (95% confidence interval (CI), 62.1%–77.7%) for the USA and 86.4% (CI, 78.4%–95.2%) for Europe [23]. Moreover, 4% and 15% of the study populations, respectively, were severely vitamin D deficient (<10 ng/mL). Similar occurrences were noted in developing countries [24].

Risk factors for suboptimal 25(OH)D include bimodal age groups (neonates, preschool children and the elderly), obesity, lack of sun exposure, inadequate diet, and presence of CKD [25,26]. Recent studies also show that air pollution can be a significant risk factor for vitamin D deficiency due to insufficient ultraviolet light [27,28,29]. Hereditary variations could also influence vitamin D status on several levels including polymorphism at the level of conversion of vitamin D precursor to 25(OH)D, 1α-hydroxylase activity, expression of VDR and variations of intracellular as well as circulating vitamin D binding protein expression. Genome-wide associate studies have showed several significant loci that can influence vitamin D status, specifically the polymorphisms of vitamin D conversion enzymes, VDR and vitamin D binding proteins [30,31,32]. Variations in these loci can influence the risk for suboptimal vitamin D.

Low circulating 25(OH)D have been shown to correlate with an elevated risk of all-cause and cardiovascular mortality. In a systematic review and meta-analysis of 73 observational cohort studies (data prior to 1 August 2013), involving 849,412 participants [23], suboptimal 25(OH)D with median (range) level of 20.7 (17.5–24.3) ng/mL was associated with cardiovascular-specific, cancer-specific and all-cause mortality with mean follow-up (range) of 6.0 (3.0–9.5) years. For each 10 ng/mL decline in circulating 25(OH)D, there was a 16% (95% CI, 8% to 23%) increase in the risk of all-cause mortality. A similar correlation was observed in several meta-analyses of prospective observational studies [21,33].

Interventional trials have generated interesting results. A prospective randomized study showed that vitamin D supplementation reduced all-cause mortality only among elderly individuals and only when vitamin D_3_ was used alone (without concomitant calcium supplementation). Vitamin D_2_ supplementation alone failed to show a significant mortality effect [23]. The lower bioavailability of vitamin D_2_ could potentially account for such an outcome [34]. Similarly, data on the relationship between cardiovascular events and vitamin D supplementation, reviewed by Wang *et al.* [35], showed that vitamin D supplementation alone, but not with calcium, significantly reduced cardiovascular events. It has been postulated that the increased calcium intake could potentially have negated the beneficial effects of vitamin D [36].

**Figure 1:**
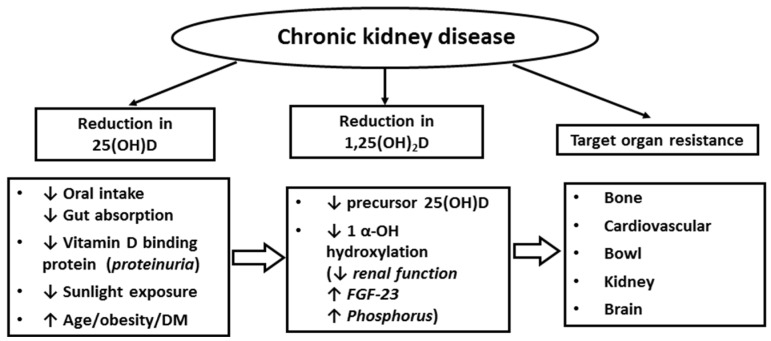
Risks of suboptimal vitamin D status in chronic kidney disease patients.

CKD patients are particularly susceptible to the development of vitamin D deficiency (Figure 1). The risk factors could be related to reduced vitamin D intake, compromised intestinal absorption, urinary loss of vitamin D binding protein, reduction in the intra-renal activity of 1α-hydroxylase leading to insufficient 1,25(OH)_2_D generation, and elevation of fibrotic growth factor 23 (FGF-23), which inhibits 1α-hydroxylase activity [37,38,39,40]. CKD patients often possess a number of additional comorbidities (advanced age, obesity, diabetes and hypertension) known to be risk factors for vitamin D deficiency [41,42]. KDIGO (Kidney Disease Improving Global Outcomes) guideline has adopted results from prior studies in CKD patients [43] and define vitamin D status similar to those in the general population (Table 1). Kim *et al.* assessed 25(OH)D status in 210 CKD patients and found that the prevalence of suboptimal vitamin D (<30 ng/mL) increased progressively with worsening CKD, 40.7% in CKD Stage 3, 61.5% in Stage 4, and 85.7% in Stage 5 [44]. A greater proportion of patients with suboptimal vitamin D had diabetes and heavy proteinuria [44]. With late stage CKD (eGFR < 15 mL/min/1.73 m^2^), uremia can cause progressive loss of tissue VDR (clearly demonstrated in the parathyroids of uremic patients [45]) and diminishing binding capacity between 1,25(OH)_2_D and VDR, leading to tissue vitamin D resistance [46,47,48]. Similar to the observations generated from the general population, multiple observational studies have demonstrated an inverse relationship between vitamin D status and all-cause mortality rates in the CKD population [49].

Vitamin D supplementation in patients with CKD is associated with reduced all-cause mortality in most observational studies [50,51], but not in randomized controlled trials [52,53,54]. Notably, most observational studies had larger numbers of participants and longer durations of observation (months to years) than those in randomized interventional trials (weeks to months). Properly sized and longer-term prospective interventional trials are needed.

## 3. Vitamin D in Brain and Neurocognitive Function

Emerging evidence suggests an important role for vitamin D in brain physiology. Vitamin D crosses the blood–brain barrier in the cerebral capillaries and enters the cerebrospinal fluid and the brain via passive diffusion and specific carriers. The concentration of 25(OH)D in the cerebrospinal fluid positively correlates with that in the serum. Vitamin D exerts its actions through VDR, which is expressed in neuronal and glial cells in almost all regions of the central nervous system. In particular, the VDR is expressed in the hippocampus, hypothalamus, cortex and subcortex, the areas essential for cognition [55,56]. 1α-hydroxylase converting 25(OH)D to 1,25(OH)_2_D is also expressed in most of these regions [57]. Vitamin D has also been found to regulate expression of tyrosine hydroxylase, the rate-limiting enzyme in the genesis of dopamine, norepinephrine and epinephrine [58].

Vitamin D promotes neuron survival [59], inhibits oxidative pathways in the brain through reducing free radical formation [60] and increases antioxidant (γ-glutamyl transpeptidase) production [61], reverses oxidative stress associated mitochondrial dysfunction [62], down-regulates l-type calcium channels expression [63] and attenuates injurious effects of excitatory neurotoxins [64,65]. Vitamin D also prevents amyloid-β accumulation by reducing the amyloid-β precursor transcription [66,67] and stimulating phagocytotic clearance of the amyloid-β peptide [55,68]. Taken together, vitamin D exerts neuroprotective and regulatory roles in the central nervous system. In line with these observations, clinical evidence shows that low serum 25(OH)D is associated with CI [3].

A diagnosis of CI can be established by demonstrating a cognitive decline from a previously attained level of functioning. It can be a decline in any of the six principle domains including complex attention, executive attention, learning and memory, language, perceptual-motor function and social cognition [69]. DSM-5 criteria put forth a set of diagnostic guidelines which are not meant to provide confirmatory diagnoses, but rather to be added to the clinical assessment of patients. Validation in clinical practice is necessary to achieve high levels of diagnostic reliability. Mini-Mental State Examination (MMSE) [70] and Modified Mini-Mental State (3MS) [71] are widely used neuropsychological instruments. They measure global cognitive function with an emphasis on memory. The Trail Making Test A focuses more on attention, and the Trail Making Test B on executive function [72]. In general, a diagnosis of CI can be made if the test scores in one or more cognitive domains fall below 1 or 2 standard deviations of those in the general population.

A meta-analyses by Balion *et al.* showed that a serum 25(OH)D level of <20 ng/mL is associated with reduced cognitive performance [73]. A further longitudinal prospective study in 858 Italian elderly individuals (>65 years) showed an increased relative risk of significant cognitive decline in those with baseline 25(OH)D levels <10 ng/mL, compared with individuals with levels ≥30 ng/mL over a 6-year period [74]. Recently, studies from the 1958 British birth cohort (at age 50 years) have shown that the presence of two APOE ε4 alleles, a known genetic risk for dementia [45], is able to modified the effects of 25(OH)D on memory function [31]. In a fully adjusted modal, 25(OH)D level was positively and progressively associated with a better memory function only in individuals carrying two APOE ε4 alleles, not in those carrying zero or one allele [31]. These results are consistent with a role for varying genetic backgrounds in the susceptibility of vitamin D deficiency associated cognitive dysfunction.

## 4. Cognitive Impairment in Patients with CKD

CKD, especially elderly CKD, population is growing [2]. CI among this segment of the population has now been increasingly recognized. A nationwide sample of U.S. community-dwelling adults (age ≥ 45 years, *n* = 30,239) participating in the REGARDS study (Reasons for Geographic and Racial Disparities in Stroke), urine albumin excretion and eGFR reduction were independently associated with CI [4,75]. Moreover, a longitudinal study of 3034 community-dwelling older adults with a mean age (SD) of 74 (3) years showed that more advanced stages of CKD were associated with progressively higher risks of CI, with odds ratio (OR) 1.32 for eGFR 45–59 mL/min/1.73 m^2^ and 2.43 for eGFR < 45 mL/min/1.73 m^2^ [76]. A meta-analysis of cross-sectional and longitudinal studies involving 54,779 participants (in the years 1980–2012) showed a positive association between cognitive decline and severity of CKD. CKD was identified as a significant and independent risk factor in the development of cognitive decline [77]. Recently, Shin *et al.* descripted a phenomenon in a population of Korea patients (*n* = 10,667), in that the presence of APOE ε4 (one and two alleles) can accelerate and amplify the degree of CI in association with albuminuria [78]. In patients on dialysis, 30%–70% develops some degrees of CI [4,76,79,80,81]. Griva *et al.* showed that two-thirds of a cohort of 145 mixed peritoneal dialysis and hemodialysis patients in London had mild-to-moderate CI in a comprehensive set of age-adjusted cognitive studies [82].

**Figure 2:**
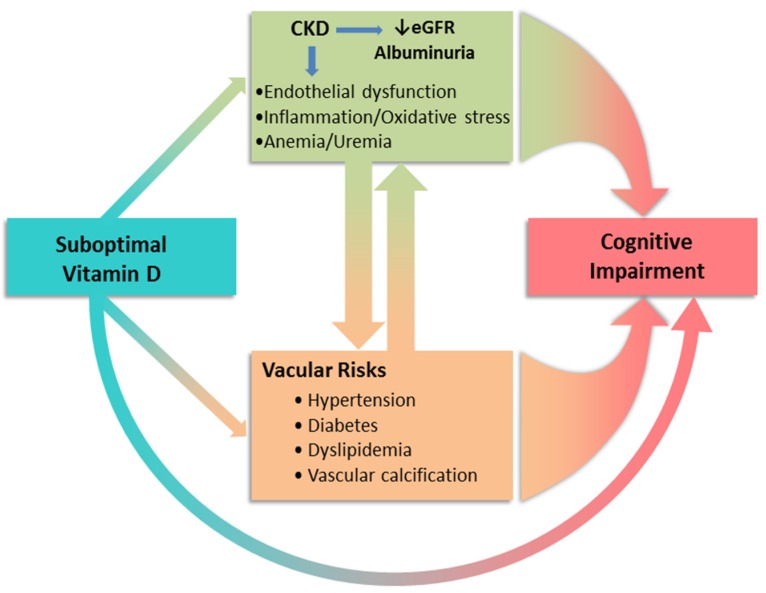
Suboptimal vitamin D status directly and indirectly contributes to the development and progression of cognitive impairment. Studies have demonstrated an association of hypovitaminosis D and diseases that raise vascular risks [85]. Endothelial dysfunction and inflammatory/uremic milieu in CKD act not only as a consequence of CKD but also through promoting vascular risk factors contributing the CKD progression.

As shown in Figure 2, in addition to worsening kidney function and albuminuria as risk factors for CI, cardiovascular risk factors, frequently co-exist with CKD such as hypertension, diabetes/metabolic syndrome and hyperlipidemia, have also been associated with the development of CI. Elevated circulating inflammatory biomarkers, characteristics in CKD [83], could further accelerate vascular diseases. Under physiological conditions, inflammatory responses remove the pathogens and initiate the healing process. In patients with CKD, a prolonged plasma half-life for some of the pro-inflammatory cytokines such as interleukin 1 and tumor necrosis factors could extend the presence of circulating cytokines and enhance inflammatory load. The inflammatory milieu in turn exacerbates endothelial dysfunction, atherogenesis and protein energy wasting, accelerating cardiovascular diseases [84].

It has been hypothesized that CI in CKD patients would have a negative clinical impact similar to that have been observed in the general population. Specifically, CI in patients with CKD is likely associated with reduced survival, increased cardiovascular events, and withdrawal of treatment for end-stage kidney disease. Thus far, studies have not been comprehensive and have generated inconsistent results [86,87,88]. More comprehensive studies involving appropriate patient source, sample size, prospective design and with detailed cognitive evaluations (including all six domains) are necessary. One such study (The COGNITIVE-HD study [89]) in dialysis patients is currently ongoing.

## 5. Potential Association between Vitamin D Deficiency and Cognitive Impairment in CKD

Few studies are available on the cognitive effects of vitamin D in patients with CKD (Table 2). Two cross sectional studies, using a similar battery of cognitive tests including MMSE/3MS and Trail Making Tests A and B, showed that patients on hemodialysis and peritoneal dialysis had significant degrees of 25(OH)D deficiency which are independently associated with worse cognitive function [90,91]. Shaffi *et al.* [90] investigated 255 hemodialysis patients in the U.S. with a mean age (SD) of 62.9 (16.9) in the years 2004 to 2012. They found that about half of the patients (49%) had vitamin D levels ≥12 and <20 ng/mL (*n* = 139), 14% had levels <12 ng/mL (*n* = 36) and only 31% had levels ≥20 ng/mL (*n* = 80). The vitamin D level ≥ 20 ng/mL was independently associated with a higher global cognitive score, and the lower vitamin D levels were associated with impaired executive function but not global function. Recently, Liu *et al.* [91] studied a population of Chinese peritoneal dialysis patients with a mean age (SD) of 53.58 (14.06) in the years 2013 to 2014. They grouped patients into two groups, vitamin D < 10 ng/mL (*n* = 163) and ≥10 ng/mL (*n* = 110). The low vitamin D level was significantly correlated with impairment of global cognitive function but not executive function.

Although the assessment batteries were similar in the two studies, there were significant differences in the patient populations (U.S. *versus* Chinese), ages (63 in U.S. patients and 54 in Chinese), dialysis modalities (hemodialysis *versus* peritoneal dialysis), baseline 25(OH)D (17.2 ng/mL *versus* 9.9 ng/mL), and study years (2004 to 2012 for hemodialysis patients and 2013 to 2014 for peritoneal dialysis patients). Patients in both studies were relatively young and should have had a lower inherent risk for low vitamin D-associated cognitive effects, as prior studies showed that elderly patients are significantly more susceptible to CI related to hypovitaminosis D [93]. Moreover, compared with hemodialysis patients, peritoneal dialysis patients tend to be more isolated as they perform their dialysis at home. Fewer social contacts could impact their global cognitive function and less outdoor sun exposure could pose risk for hypovitaminosis D. In both studies, a fraction of patients were incident-dialysis patients and can be loosely considered CKD patients. Despite the multiple differences, the overall results of the two studies suggest vitamin D deficiency can be an independent risk factor for CI.

Not all studies, however, have shown a positive association. Jovanovich *et al.* [92] conducted a study in non-dialysis and dialysis CKD patients at 36 Veterans Affairs Medical Centers in the years 2001 to 2006. They found no association between cognitive function and vitamin D levels. There were, however, several major limitations. The blood samples for vitamin D measurements between December 2002 and January 2004, several years prior to the cognitive tests conducted in June 2005 to July 2006. It is unknown whether the vitamin D levels in the cognitive study period were remained the same as those in the earlier years. The tests were conducted via phone calls (each lasting more than 20 min), which might have resulted a selection bias. Patients not feeling physically well, or with some degree of CI, might not have been able to tolerate a 20 plus min phone conversation. These patients likely would not have been included in the study. Thus, the selection of the participants could have been biased toward a healthier patient population and null hypothesis. The study used a different battery of instruments for measuring the cognitive capacity. Thus, it is hard to compare this study with the studies by Shaffi *et al.* [90] and Liu *et al.* [91].

Taken together, given the neuroprotective and other multiple beneficial effects of vitamin D, it is tempting to assume that vitamin D deficiency could potentially contribute to CI in patients with CKD. The studies reported above, however, offer only limited information on a possible association between low vitamin D and risk for some aspects of cognitive function. Differences in study protocols and small sample sizes precluded drawing any conclusion, which underscores a critical need of further investigation. Prospective randomized controlled study with proper sample size and more nuanced cognitive evaluations would provide a much clearer picture and a causal relation between a low vitamin D and CI in CKD. At least one such study (NCT01229878) [94] is ongoing. The results from the study are eagerly awaited.

## 6. Implications for Clinical Practice

CI can lead to a global and functional deterioration that interferes with activities of daily living and adherence to prescribed diet and medications, which, in turn, can be deleterious as diet and medications are critical parts of the CKD management to control hypertension, diabetes, volume status and mineral and bone disorders. These detrimental effects can lead to patient morbidity and mortality as well as to increased utilization of healthcare resources. Neuropsychological assessment should, therefore, be considered as a part of CKD care, especially given CI can occur in all stages of CKD [95].

Several well-established instruments are clinically available. For initial screening, the abbreviated Six-Item Assessment of Cognitive Function derived from 3MS, could be useful. It takes less than 5 min and has been shown to be effective in identifying CKD patients with CI [75,96]. Following the screening, several more extensive tools, such as detailed versions of MMSE, 3MS, Trail Making Test A and B, and the Montreal Cognitive Assessment tool (MoCA) could be administered to fine tune specific domains of cognitive defects [72,97].

Given the well-established beneficial effects of vitamin D on musculoskeletal health and all-cause mortality, timely identification of hypovitaminosis D and restoration of adequate vitamin D status in CKD would likely exert beneficial effects. Moreover, vitamin D insufficiency and deficiency may represent a reversible risk component for the development and progression of CI. This assumption is supported by the fact that adequate vitamin D status can directly and indirectly protect patients from CI by attenuating cardiovascular risk factors, such as insulin resistance, endothelial dysfunction, RAS hyperactivation, reactive oxygen specie formation and systemic inflammation [98] and by neuroprotective effects of vitamin D. Fortunately, vitamin D status can be screened easily and repleted. We suggest screening vitamin D status periodically for CKD patients and supplementing if appropriate. Moreover, different ethnicity, culture, clothing habits, outdoor activity, dietary choices and genetic background (APOE ε status) should all be taken into consideration in determining the timing and frequency of evaluation and management of vitamin D status. Current practice is to maintain serum 25(OH)D at an optimal range of >30 to 80 ng/mL in patients with CKD [22].

Adverse effects of vitamin D supplementation are rare. Hypercalcemia is the major adverse effect and is extremely rare with the current 25(OH)D target [99]. In rodent experiments, extreme 25(OH)D elevation to approximately 330 ng/mL has been shown to give rise to kidney tubular interstitial injury due to altered macrophage phenotype [100]. Such a high level would not be attainable in humans with the vitamin D supplementation regimens used in the current practice. A recent study reported in one of 197 CKD patients who were treated with cholecalciferol 1000 IU/day developed a mild asymptomatic calcium elevation of 10.5 mg/dL [44]. A pilot trial using oral cholecalciferol 50,000 IU weekly for six months for hemodialysis patients with suboptimal baseline vitamin D status is ongoing [94]. The results could provide valuable information on the tolerability of such a supplementation regimen. We look forward to future randomized controlled trials to shed light on the tolerability and effects of vitamin D repletion, specifically on cognitive function in patients with CKD. Meanwhile, integrated care bringing together clinicians, nurses, nutritionists and clinician researchers would likely benefit CKD patients through effective prevention and treatment of hypovitaminosis D, thereby optimize cognitive function and patient outcomes.

## 7. Conclusions

CI in patients with kidney dysfunction has received much attention in the last decade. Emerging evidence suggests a role for a reduced circulating 25(OH)D (insufficiency and deficiency) in CKD, through CKD-dependent and CKD-independent mechanisms, on the development and progression of cognitive decline. CKD patients show a disproportionately high incidence of hypovitaminosis D. The combination of vitamin D deficiency and CKD may exert a synergistic and deleterious effect on cognition. As improving cognitive function could be one potential mechanism through which vitamin D exerts its beneficial effects in CKD, vitamin D repletion holds promise. Further well-designed randomized controlled trials are, however, required to clarify whether vitamin D indeed exert clinically relevant long-term beneficial effects on cognitive function in CKD.

## Figures and Tables

**Table 1:** Classification of Vitamin D Status by 25(OH)D concentration.

Classification	25(OH)D Concentration
Optimal	>30–80 ng/mL
Suboptimal	
-Insufficient	20–30 ng/mL
-Deficient	<20 ng/mL

Note: ng/mL can be converted to nmol/L by multiplying 2.5.

**Table 2:** Comparison of the three studies on the relationship between vitamin D deficiency and cognitive impairment in CKD patients.

	Shaffi *et al*. 2013 [90]	Liu *et al.* 2015 [91]	Jovanovich *et al.* 2014 [92]
	*N* = 255	*N* = 273	*N* = 605
Database	2004–2012	2013–2014	2001–2004 blood test 2005–2006 cognitive test
Units	5 dialysis clinic units and 1 hospital-based unit (USA)	2 hospitals/PD centers (China)	36 medical centers (USA)
Dialysis modality	HD	PD	CKD + ESRD/HD (247 + 358)
Age (mean ± SD, years)	62.9 ± 16.9	53.6 ± 14.1	67 ± 12
Male (%)	140 (54.9)	136 (49.8)	595 (98.3)
Hypertension (%)	231 (90.6)	-	585 (96.7)
Diabetes mellitus (%)	118 (46.3)	73 (26.7)	299 (49.4)
Dialysis duration (range, months)	15 (7–35)	26.8 (10.9–55.4)	-
Serum 25(OH)D (mean ± SD, ng/mL)	17.2 ± 7.4	9.9 ± 3.7	Median 18 (range12–25)
25(OH)D cut-off (patients’ number)	<12 ng/mL (36) 12 to <20 ng/mL (139) ≥20 ng/mL (80)	<10 ng/mL (163) ≥10 ng/mL (110)	<13 ng/mL 13–22 ng/mL
Cognitive tests	MMSE , WMS-III Word List Learning Subtest, WAIS-III Block Design and subtests, WAIS-III Digit Symbol Coding, TMT A, TMT B, Digit Span, Mental Alternation Test, COWAT	3MS, TMT A, TMT B, RBANS sub-tests	TICSm
Main cognitive impairment associated with 25(OH)D concentration	Executive function	Global cognitive function	-

PD, peritoneal dialysis; HD, hemodialysis; CKD, chronic kidney disease; ESRD, end stage renal disease; SD, standard deviation; MMSE, Mini-Mental State Examination; WMS-III, Wechsler Memory Scale-III; WAIS-III Wechsler Adult Intelligence Scale-III, 3MS, Modified Mini-Mental State Examination; TMT A, Trail Making Tests A; TMT B, Trail Making Tests B; COWAT, Controlled Oral Word Association Test; RBANS, Repeatable Battery for the Assessment of Neuropsychological Status; TICSm, Telephone Interview for Cognitive Status-modified.

## Abbreviations

CKD	chronic kidney disease
CI	cognitive impairment
VDR	vitamin D receptor
KDIGO	Kidney Disease Improving Global Outcomes
eGFR	estimated glomerular filtration rate
MMSE	Mini-Mental State Examination
3MS	Modified Mini-Mental State Exam

## References

[1] Holick M.F. (2007). Vitamin d deficiency. N. Engl. J. Med..

[2] Coresh J., Selvin E., Stevens L.A., Manzi J., Kusek J.W., Eggers P., Van Lente F., Levey A.S. (2007). Prevalence of chronic kidney disease in the united states. JAMA.

[3] Etgen T., Sander D., Bickel H., Sander K., Forstl H. (2012). Vitamin D deficiency, cognitive impairment and dementia: A systematic review and meta-analysis. Dement. Geriatr. Cogn. Disord..

[4] Kurella Tamura M., Wadley V., Yaffe K., McClure L.A., Howard G., Go R., Allman R.M., Warnock D.G., McClellan W. (2008). Kidney function and cognitive impairment in us adults: The reasons for geographic and racial differences in stroke (REGARDS) study. Am. J. Kidney Dis..

[5] Hewison M., Zehnder D., Chakraverty R., Adams J.S. (2004). Vitamin D and barrier function: A novel role for extra-renal 1 alpha-hydroxylase. Mol. Cell. Endocrinol..

[6] Jones G. (2007). Expanding role for vitamin D in chronic kidney disease: Importance of blood 25-OH-D levels and extra-renal 1alpha-hydroxylase in the classical and nonclassical actions of 1alpha,25-dihydroxyvitamin D(3). Semin. Dial..

[7] Al-Badr W., Martin K.J. (2008). Vitamin D and kidney disease. Clin. J. Am. Soc. Nephrol..

[8] Wei R., Christakos S. (2015). Mechanisms underlying the regulation of innate and adaptive immunity by vitamin D. Nutrients.

[9] Lowry M.B., Guo C., Borregaard N., Gombart A.F. (2014). Regulation of the human cathelicidin antimicrobial peptide gene by 1alpha,25-dihydroxyvitamin D3 in primary immune cells. J. Steroid Biochem. Mol. Biol..

[10] Heaney R.P. (2008). Vitamin D in health and disease. Clin. J. Am. Soc. Nephrol..

[11] Zipitis C.S., Akobeng A.K. (2008). Vitamin D supplementation in early childhood and risk of type 1 diabetes: A systematic review and meta-analysis. Arch. Dis. Child..

[12] Ranganathan P., Khalatbari S., Yalavarthi S., Marder W., Brook R., Kaplan M.J. (2013). Vitamin D deficiency, interleukin 17, and vascular function in rheumatoid arthritis. J. Rheumatol..

[13] Summerday N.M., Brown S.J., Allington D.R., Rivey M.P. (2012). Vitamin D and multiple sclerosis: Review of a possible association. J. Pharm. Pract..

[14] Runia T.F., Hop W.C., de Rijke Y.B., Buljevac D., Hintzen R.Q. (2012). Lower serum vitamin D levels are associated with a higher relapse risk in multiple sclerosis. Neurology.

[15] Martineau A.R., James W.Y., Hooper R.L., Barnes N.C., Jolliffe D.A., Greiller C.L., Islam K., McLaughlin D., Bhowmik A., Timms P.M. (2015). Vitamin D3 supplementation in patients with chronic obstructive pulmonary disease (VIDICO): A multicentre, double-blind, randomised controlled trial. Lancet Respir. Med..

[16] Arnedo-Pena A., Juan-Cerdan J.V., Romeu-Garcia M.A., Garcia-Ferrer D., Holguin-Gomez R., Iborra-Millet J., Pardo-Serrano F. (2015). Vitamin D status and incidence of tuberculosis infection conversion in contacts of pulmonary tuberculosis patients: A prospective cohort study. Epidemiol. Infect..

[17] Chen G.C., Zhang Z.L., Wan Z., Wang L., Weber P., Eggersdorfer M., Qin L.Q., Zhang W. (2015). Circulating 25-hydroxyvitamin D and risk of lung cancer: A dose-response meta-analysis. Cancer Causes Control.

[18] Chen P., Li M., Gu X., Liu Y., Li X., Li C., Wang Y., Xie D., Wang F., Yu C. (2013). Higher blood 25(OH)D level may reduce the breast cancer risk: Evidence from a Chinese population based case-control study and meta-analysis of the observational studies. PLoS ONE.

[19] Hollis B.W. (2007). Assessment of circulating 25(OH)D and 1,25(OH)2D: Emergence as clinically important diagnostic tools. Nutr. Rev..

[20] Hollis B.W. (2008). Assessment of vitamin D status and definition of a normal circulating range of 25-hydroxyvitamin D. Curr. Opin. Endocrinol. Diabetes Obes..

[21] Zittermann A., Iodice S., Pilz S., Grant W.B., Bagnardi V., Gandini S. (2012). Vitamin D deficiency and mortality risk in the general population: A meta-analysis of prospective cohort studies. Am. J. Clin. Nutr..

[22] Chonchol M., Kendrick J., Targher G. (2011). Extra-skeletal effects of vitamin D deficiency in chronic kidney disease. Ann. Med..

[23] Chowdhury R., Kunutsor S., Vitezova A., Oliver-Williams C., Chowdhury S., Kiefte-de-Jong J.C., Khan H., Baena C.P., Prabhakaran D., Hoshen M.B. (2014). Vitamin D and risk of cause specific death: Systematic review and meta-analysis of observational cohort and randomised intervention studies. BMJ.

[24] Arabi A., El Rassi R., El-Hajj Fuleihan G. (2010). Hypovitaminosis D in developing countries-prevalence, risk factors and outcomes. Nat. Rev. Endocrinol..

[25] Bassil D., Rahme M., Hoteit M., Fuleihan Gel H. (2013). Hypovitaminosis D in the Middle East and North Africa: Prevalence, risk factors and impact on outcomes. Dermatoendocrinology.

[26] Holick M.F., Binkley N.C., Bischoff-Ferrari H.A., Gordon C.M., Hanley D.A., Heaney R.P., Murad M.H., Weaver C.M., Endocrine S. (2011). Evaluation, treatment, and prevention of vitamin D deficiency: An endocrine society clinical practice guideline. J. Clin. Endocrinol. Metab..

[27] Kelishadi R., Moeini R., Poursafa P., Farajian S., Yousefy H., Okhovat-Souraki A.A. (2014). Independent association between air pollutants and vitamin D deficiency in young children in isfahan, Iran. Paediatr. Int. Child Health.

[28] Hosseinpanah F., Pour S.H., Heibatollahi M., Moghbel N., Asefzade S., Azizi F. (2010). The effects of air pollution on vitamin D status in healthy women: A cross sectional study. BMC Public Health.

[29] Calderon-Garciduenas L., Franco-Lira M., D’Angiulli A., Rodriguez-Diaz J., Blaurock-Busch E., Busch Y., Chao C.K., Thompson C., Mukherjee P.S., Torres-Jardon R. (2015). Mexico City normal weight children exposed to high concentrations of ambient PM 2.5 show high blood leptin and endothelin-1, vitamin D deficiency, and food reward hormone dysregulation *versus* low pollution controls. Relevance for obesity and Alzheimer disease. Environ. Res..

[30] Ahn J., Yu K., Stolzenberg-Solomon R., Simon K.C., McCullough M.L., Gallicchio L., Jacobs E.J., Ascherio A., Helzlsouer K., Jacobs K.B. (2010). Genome-wide association study of circulating vitamin D levels. Hum. Mol. Genet..

[31] Shea M.K., Benjamin E.J., Dupuis J., Massaro J.M., Jacques P.F., D’Agostino R.B., Ordovas J.M., O’Donnell C.J., Dawson-Hughes B., Vasan R.S. (2009). Genetic and non-genetic correlates of vitamins K and D. Eur. J. Clin. Nutr..

[32] Wang T.J., Zhang F., Richards J.B., Kestenbaum B., van Meurs J.B., Berry D., Kiel D.P., Streeten E.A., Ohlsson C., Koller D.L. (2010). Common genetic determinants of vitamin D insufficiency: A genome-wide association study. Lancet.

[33] Schöttker B., Jorde R., Peasey A., Thorand B., Jansen E.H., Groot L., Streppel M., Gardiner J., Ordonez-Mena J.M., Perna L. (2014). Vitamin D and mortality: Meta-analysis of individual participant data from a large consortium of cohort studies from Europe and the United States. BMJ.

[34] Borel P., Caillaud D., Cano N.J. (2015). Vitamin D bioavailability: State of the art. Crit. Rev. Food Sci. Nutr..

[35] Wang L., Manson J.E., Song Y., Sesso H.D. (2010). Systematic review: Vitamin D and calcium supplementation in prevention of cardiovascular events. Ann. Intern. Med..

[36] Rossom R.C., Espeland M.A., Manson J.E., Dysken M.W., Johnson K.C., Lane D.S., LeBlanc E.S., Lederle F.A., Masaki K.H., Margolis K.L. (2012). Calcium and vitamin D supplementation and cognitive impairment in the women’s health initiative. J. Am. Geriatr. Soc..

[37] Echida Y., Mochizuki T., Uchida K., Tsuchiya K., Nitta K. (2012). Risk factors for vitamin D deficiency in patients with chronic kidney disease. Intern. Med..

[38] Krajisnik T., Bjorklund P., Marsell R., Ljunggren O., Akerstrom G., Jonsson K.B., Westin G., Larsson T.E. (2007). Fibroblast growth factor-23 regulates parathyroid hormone and 1alpha-hydroxylase expression in cultured bovine parathyroid cells. J. Endocrinol..

[39] Hasegawa H., Nagano N., Urakawa I., Yamazaki Y., Iijima K., Fujita T., Yamashita T., Fukumoto S., Shimada T. (2010). Direct evidence for a causative role of FGF23 in the abnormal renal phosphate handling and vitamin D metabolism in rats with early-stage chronic kidney disease. Kidney Int..

[40] Sato K.A., Gray R.W., Lemann J. (1982). Urinary excretion of 25-hydroxyvitamin D in health and the nephrotic syndrome. J. Lab. Clin. Med..

[41] Jacob A.I., Sallman A., Santiz Z., Hollis B.W. (1984). Defective photoproduction of cholecalciferol in normal and uremic humans. J. Nutr..

[42] Tsiaras W.G., Weinstock M.A. (2011). Factors influencing vitamin D status. Acta Derm. Venereol..

[43] Kidney Disease: Improving Global Outcomes (KDIGO) CKD-MBD Work Group (2009). KDIGO clinical practice guideline for the diagnosis, evaluation, prevention, and treatment of chronic kidney disease-mineral and bone disorder (CKD-MBD). Kidney Int. Suppl..

[44] Kim S.M., Choi H.J., Lee J.P., Kim D.K., Oh Y.K., Kim Y.S., Lim C.S. (2014). Prevalence of vitamin D deficiency and effects of supplementation with cholecalciferol in patients with chronic kidney disease. J. Ren. Nutr..

[45] Fukuda N., Tanaka H., Tominaga Y., Fukagawa M., Kurokawa K., Seino Y. (1993). Decreased 1,25-dihydroxyvitamin D3 receptor density is associated with a more severe form of parathyroid hyperplasia in chronic uremic patients. J. Clin. Investig..

[46] Nigwekar S.U., Bhan I., Thadhani R. (2012). Ergocalciferol and cholecalciferol in CKD. Am. J. Kidney Dis..

[47] Hsu C.H., Patel S.R. (1995). Altered vitamin D metabolism and receptor interaction with the target genes in renal failure: Calcitriol receptor interaction with its target gene in renal failure. Curr. Opin. Nephrol. Hypertens..

[48] Patel S.R., Ke H.Q., Vanholder R., Koenig R.J., Hsu C.H. (1995). Inhibition of calcitriol receptor binding to vitamin D response elements by uremic toxins. J. Clin. Investig..

[49] Pilz S., Iodice S., Zittermann A., Grant W.B., Gandini S. (2011). Vitamin D status and mortality risk in CKD: A meta-analysis of prospective studies. Am. J. Kidney Dis..

[50] Zheng Z., Shi H., Jia J., Li D., Lin S. (2013). Vitamin D supplementation and mortality risk in chronic kidney disease: A meta-analysis of 20 observational studies. BMC Nephrol..

[51] Duranton F., Rodriguez-Ortiz M.E., Duny Y., Rodriguez M., Daures J.P., Argiles A. (2013). Vitamin D treatment and mortality in chronic kidney disease: A systematic review and meta-analysis. Am. J. Nephrol..

[52] Xu L., Wan X., Huang Z., Zeng F., Wei G., Fang D., Deng W., Li Y. (2013). Impact of vitamin D on chronic kidney diseases in non-dialysis patients: A meta-analysis of randomized controlled trials. PLoS ONE.

[53] Palmer S.C., McGregor D.O., Macaskill P., Craig J.C., Elder G.J., Strippoli G.F. (2007). Meta-analysis: Vitamin D compounds in chronic kidney disease. Ann. Intern. Med..

[54] Mann M.C., Hobbs A.J., Hemmelgarn B.R., Roberts D.J., Ahmed S.B., Rabi D.M. (2015). Effect of oral vitamin D analogs on mortality and cardiovascular outcomes among adults with chronic kidney disease: A meta-analysis. Clin. Kidney J..

[55] Annweiler C., Dursun E., Feron F., Gezen-Ak D., Kalueff A.V., Littlejohns T., Llewellyn D.J., Millet P., Scott T., Tucker K.L. (2015). “Vitamin D and cognition in older adults”: Updated international recommendations. J. Intern. Med..

[56] Prufer K., Veenstra T.D., Jirikowski G.F., Kumar R. (1999). Distribution of 1,25-dihydroxyvitamin D3 receptor immunoreactivity in the rat brain and spinal cord. J. Chem. Neuroanat..

[57] Eyles D.W., Smith S., Kinobe R., Hewison M., McGrath J.J. (2005). Distribution of the vitamin D receptor and 1 alpha-hydroxylase in human brain. J. Chem. Neuroanat..

[58] Garcion E., Wion-Barbot N., Montero-Menei C.N., Berger F., Wion D. (2002). New clues about vitamin D functions in the nervous system. Trends Endocrinol. Metab..

[59] Smith M.P., Fletcher-Turner A., Yurek D.M., Cass W.A. (2006). Calcitriol protection against dopamine loss induced by intracerebroventricular administration of 6-hydroxydopamine. Neurochem. Res..

[60] Garcion E., Nataf S., Berod A., Darcy F., Brachet P. (1997). 1,25-dihydroxyvitamin D3 inhibits the expression of inducible nitric oxide synthase in rat central nervous system during experimental allergic encephalomyelitis. Brain Res. Mol. Brain Res..

[61] Baas D., Prufer K., Ittel M.E., Kuchler-Bopp S., Labourdette G., Sarlieve L.L., Brachet P. (2000). Rat oligodendrocytes express the vitamin D(3) receptor and respond to 1,25-dihydroxyvitamin D(3). Glia.

[62] Li L., Prabhakaran K., Zhang X., Zhang L., Liu H., Borowitz J.L., Isom G.E. (2008). 1alpha,25-dihydroxyvitamin D3 attenuates cyanide-induced neurotoxicity by inhibiting uncoupling protein-2 up-regulation. J. Neurosci. Res..

[63] Brewer L.D., Thibault V., Chen K.C., Langub M.C., Landfield P.W., Porter N.M. (2001). Vitamin D hormone confers neuroprotection in parallel with downregulation of l-type calcium channel expression in hippocampal neurons. J. Neurosci..

[64] Taniura H., Ito M., Sanada N., Kuramoto N., Ohno Y., Nakamichi N., Yoneda Y. (2006). Chronic vitamin D3 treatment protects against neurotoxicity by glutamate in association with upregulation of vitamin D receptor mrna expression in cultured rat cortical neurons. J. Neurosci. Res..

[65] Wang J.Y., Wu J.N., Cherng T.L., Hoffer B.J., Chen H.H., Borlongan C.V., Wang Y. (2001). Vitamin D(3) attenuates 6-hydroxydopamine-induced neurotoxicity in rats. Brain Res..

[66] Wang L., Hara K., Van Baaren J.M., Price J.C., Beecham G.W., Gallins P.J., Whitehead P.L., Wang G., Lu C., Slifer M.A. (2012). Vitamin D receptor and alzheimer’s disease: A genetic and functional study. Neurobiol. Aging.

[67] Grimm M.O., Lehmann J., Mett J., Zimmer V.C., Grosgen S., Stahlmann C.P., Hundsdorfer B., Haupenthal V.J., Rothhaar T.L., Herr C. (2014). Impact of vitamin D on amyloid precursor protein processing and amyloid-beta peptide degradation in Alzheimer’s disease. Neurodegener. Dis..

[68] Almeras L., Eyles D., Benech P., Laffite D., Villard C., Patatian A., Boucraut J., Mackay-Sim A., McGrath J., Feron F. (2007). Developmental vitamin D deficiency alters brain protein expression in the adult rat: Implications for neuropsychiatric disorders. Proteomics.

[69] Bartley J. (2010). Vitamin D: Emerging roles in infection and immunity. Expert Rev. Anti-Infect. Ther..

[70] Maddock J., Cavadino A., Power C., Hypponen E. (2015). 25-hydroxyvitamin D, APOE varepsilon4 genotype and cognitive function: Findings from the 1958 British birth cohort. Eur. J. Clin. Nutr..

[71] Teng E.L., Chui H.C. (1987). The Modified Mini-Mental State (3MS) examination. J. Clin. Psychiatry.

[72] Schneider S.M., Kielstein J.T., Braverman J., Novak M. (2015). Cognitive function in patients with chronic kidney disease: Challenges in neuropsychological assessments. Semin. Nephrol..

[73] Balion C., Griffith L.E., Strifler L., Henderson M., Patterson C., Heckman G., Llewellyn D.J., Raina P. (2012). Vitamin D, cognition, and dementia: A systematic review and meta-analysis. Neurology.

[74] Llewellyn D.J., Lang I.A., Langa K.M., Muniz-Terrera G., Phillips C.L., Cherubini A., Ferrucci L., Melzer D. (2010). Vitamin D and risk of cognitive decline in elderly persons. Arch. Intern. Med..

[75] Kurella Tamura M., Muntner P., Wadley V., Cushman M., Zakai N.A., Bradbury B.D., Kissela B., Unverzagt F., Howard G., Warnock D. (2011). Albuminuria, kidney function, and the incidence of cognitive impairment among adults in the United States. Am. J. Kidney Dis..

[76] Kurella M., Chertow G.M., Fried L.F., Cummings S.R., Harris T., Simonsick E., Satterfield S., Ayonayon H., Yaffe K. (2005). Chronic kidney disease and cognitive impairment in the elderly: The health, aging, and body composition study. J. Am. Soc. Nephrol..

[77] Etgen T., Chonchol M., Forstl H., Sander D. (2012). Chronic kidney disease and cognitive impairment: A systematic review and meta-analysis. Am. J. Nephrol..

[78] Shin M.H., Kweon S.S., Choi J.S., Lee Y.H., Nam H.S., Park K.S., Kim H.N., Oh S.Y., Jeong S.K. (2014). A disease modification effect of APOE E4 on the association between urinary albumin excretion and cognition in Korean adults. Dis. Markers.

[79] Kurella M., Chertow G.M., Luan J., Yaffe K. (2004). Cognitive impairment in chronic kidney disease. J. Am. Geriatr. Soc..

[80] Kurella Tamura M., Larive B., Unruh M.L., Stokes J.B., Nissenson A., Mehta R.L., Chertow G.M. (2010). Prevalence and correlates of cognitive impairment in hemodialysis patients: The frequent hemodialysis network trials. Clin. J. Am. Soc. Nephrol..

[81] Yaffe K., Ackerson L., Kurella Tamura M., Le Blanc P., Kusek J.W., Sehgal A.R., Cohen D., Anderson C., Appel L., Desalvo K. (2010). Chronic kidney disease and cognitive function in older adults: Findings from the chronic renal insufficiency cohort cognitive study. J. Am. Geriatr. Soc..

[82] Griva K., Stygall J., Hankins M., Davenport A., Harrison M., Newman S.P. (2010). Cognitive impairment and 7-year mortality in dialysis patients. Am. J. Kidney Dis..

[83] Gupta J., Mitra N., Kanetsky P.A., Devaney J., Wing M.R., Reilly M., Shah V.O., Balakrishnan V.S., Guzman N.J., Girndt M. (2012). Association between albuminuria, kidney function, and inflammatory biomarker profile in CKD in CRIC. Clin. J. Am. Soc. Nephrol..

[84] Stenvinkel P., Heimburger O., Paultre F., Diczfalusy U., Wang T., Berglund L., Jogestrand T. (1999). Strong association between malnutrition, inflammation, and atherosclerosis in chronic renal failure. Kidney Int..

[85] Anagnostis P., Athyros V.G., Adamidou F., Florentin M., Karagiannis A. (2010). Vitamin D and cardiovascular disease: A novel agent for reducing cardiovascular risk?. Curr. Vasc. Pharmacol..

[86] Kurella M., Mapes D.L., Port F.K., Chertow G.M. (2006). Correlates and outcomes of dementia among dialysis patients: The dialysis outcomes and practice patterns study. Nephrol. Dial. Transplant..

[87] Sarnak M.J., Tighiouart H., Scott T.M., Lou K.V., Sorensen E.P., Giang L.M., Drew D.A., Shaffi K., Strom J.A., Singh A.K. (2013). Frequency of and risk factors for poor cognitive performance in hemodialysis patients. Neurology.

[88] Kurella Tamura M., Yaffe K., Hsu C.Y., Yang J., Sozio S., Fischer M., Chen J., Ojo A., DeLuca J., Xie D. (2016). Cognitive impairment and progression of CKD. Am. J. Kidney Dis..

[89] Palmer S.C., Ruospo M., Barulli M.R., Iurillo A., Saglimbene V., Natale P., Gargano L., Murgo A.M., Loy C., van Zwieten A. (2015). COGNITIVE-HD study: Protocol of an observational study of neurocognitive functioning and association with clinical outcomes in adults with end-stage kidney disease treated with haemodialysis. BMJ Open.

[90] Shaffi K., Tighiouart H., Scott T., Lou K., Drew D., Weiner D., Sarnak M. (2013). Low 25-hydroxyvitamin D levels and cognitive impairment in hemodialysis patients. Clin. J. Am. Soc. Nephrol..

[91] Liu G.L., Pi H.C., Hao L., Li D.D., Wu Y.G., Dong J. (2015). Vitamin D status is an independent risk factor for global cognitive impairment in peritoneal dialysis patients. PLoS ONE.

[92] Jovanovich A.J., Chonchol M., Brady C.B., Kaufman J.D., Kendrick J., Cheung A.K., Jablonski K.L. (2014). 25-vitamin D, 1,25-vitamin D, parathyroid hormone, fibroblast growth factor-23 and cognitive function in men with advanced CKD: A veteran population. Clin. Nephrol..

[93] Miller J.W., Harvey D.J., Beckett L.A., Green R., Farias S.T., Reed B.R., Olichney J.M., Mungas D.M., DeCarli C. (2015). Vitamin D status and rates of cognitive decline in a multiethnic cohort of older adults. JAMA Neurol..

[94] National Institutes of Health: Vitamin D Supplementation on Physical and Cognitive Function-Pilot Study. https://clinicaltrials.gov/ct2/show/NCT01229878.

[95] Romijn M.D., van Marum R.J., Emmelot-Vonk M.H., Verhaar H.J., Koek H.L. (2015). Mild chronic kidney disease is associated with cognitive function in patients presenting at a memory clinic. Int. J. Geriatr. Psychiatry.

[96] Callahan C.M., Unverzagt F.W., Hui S.L., Perkins A.J., Hendrie H.C. (2002). Six-item screener to identify cognitive impairment among potential subjects for clinical research. Med. Care.

[97] Tiffin-Richards F.E., Costa A.S., Holschbach B., Frank R.D., Vassiliadou A., Kruger T., Kuckuck K., Gross T., Eitner F., Floege J. (2014). The montreal cognitive assessment (MOCA)—A sensitive screening instrument for detecting cognitive impairment in chronic hemodialysis patients. PLoS ONE.

[98] Siew E.D., Ikizler T.A. (2008). Determinants of insulin resistance and its effects on protein metabolism in patients with advanced chronic kidney disease. Contrib. Nephrol..

[99] Glade M.J. (2012). A 21st century evaluation of the safety of oral vitamin D. Nutrition.

[100] Kusunoki Y., Matsui I., Hamano T., Shimomura A., Mori D., Yonemoto S., Takabatake Y., Tsubakihara Y., St-Arnaud R., Isaka Y. (2015). Excess 25-hydroxyvitamin D3 exacerbates tubulointerstitial injury in mice by modulating macrophage phenotype. Kidney Int..

